# Methods of Purification and Application Procedures of Alpha1 Antitrypsin: A Long-Lasting History

**DOI:** 10.3390/molecules25174014

**Published:** 2020-09-02

**Authors:** Simona Viglio, Paolo Iadarola, Maura D’Amato, Jan Stolk

**Affiliations:** 1 Department of Molecular Medicine, University of Pavia, 27100 Pavia, Italy; simona.viglio@unipv.it (S.V.); maura.damato90@gmail.com (M.D.); 2 Department of Biology and Biotechnologies “L.Spallanzani”, University of Pavia, 27100 Pavia, Italy; 3 Department of Pulmonology, Leiden University Medical Center, 2333 Leiden, The Netherlands; J.Stolk@lumc.nl

**Keywords:** alpha1-antitrypsin, AAT, alpha1-antitrypsin deficiency, AATD, protein purification, AAT replacement therapy

## Abstract

The aim of the present report is to review the literature addressing the methods developed for the purification of alpha1-antitrypsin (AAT) from the 1950s to the present. AAT is a glycoprotein whose main function is to protect tissues from human neutrophil elastase (HNE) and other proteases released by neutrophils during an inflammatory state. The lack of this inhibitor in human serum is responsible for the onset of alpha1-antitrypsin deficiency (AATD), which is a severe genetic disorder that affects lungs in adults and for which there is currently no cure. Being used, under special circumstances, as a medical treatment of AATD in the so-called “replacement” therapy (consisting in the intravenous infusion of the missing protein), AAT is a molecule with a lot of therapeutic importance. For this reason, interest in AAT purification from human plasma or its production in a recombinant version has grown considerably in recent years. This article retraces all technological advances that allowed the manufacturers to move from a few micrograms of partially purified AAT to several grams of highly purified protein. Moreover, the chronic augmentation and maintenance therapy in individuals with emphysema due to congenital AAT deficiency (current applications in the clinical setting) is also presented.

## 1. Introduction

Alpha1-antitrypsin (AAT) is an acute-phase glycoprotein whose main function is to protect tissues from the enzymes of inflammatory cells, especially human neutrophil elastase (HNE) released by neutrophils during an inflammatory state [1,2,3,4,5,6]. Besides its high specificity for HNE, AAT has also the ability to neutralize several other proteases including cathepsin G (Cat G), proteinase 3, metalloproteases, cysteine proteases, and aspartic proteases [7,8,9,10].

**Figure 1:**
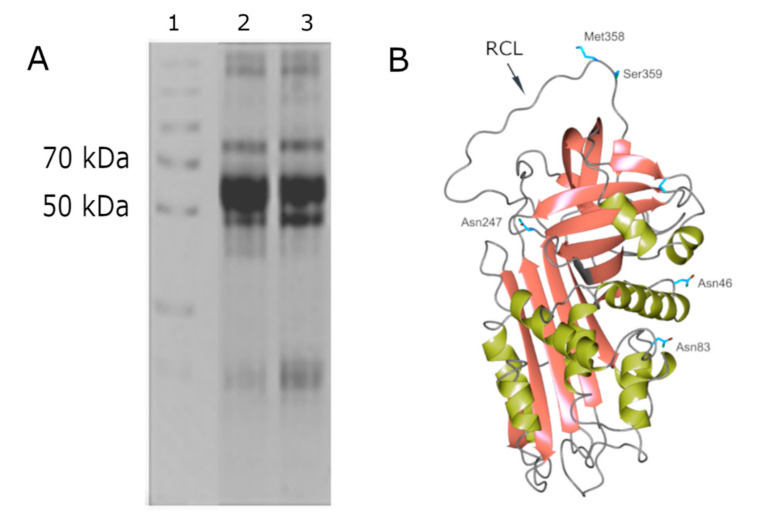
(**A**) 12.5% SDS-PAGE showing the protein profile obtained from bronchoalveolar lavage fluid (BALF) samples from patients affected by Bronchilolitis Obliterans Syndrome (BOS). Alpha1-antitrypsin (AAT, database entry ID P01009 according to UniProt) and the AAT–HNE (human neutrophil elastase) complex are represented as single bands with an approximate Mr of 52 kDa and 80 kDa, respectively (unpublished data from our laboratory). Lane 1: standard proteins with known Mrs. (**B**) 3D structure of AAT. The beta sheets are represented in red while the alpha-helices are represented in green. The reactive center loop (RCL) in the upper pole of the molecule shows the P1–P1′ residues (Met358 and Ser359) recognized by HNE. Asn46, Asn83 and Asn247 are the residues to which the three carbohydrate side chains are linked. The molecular weight (MW) of AAT and its isoelectric point (pI) are 52 kDa and 5.1, respectively. The protein is synthesized in the liver and has a half-life of 4–5 days in healthy conditions.

AAT is a 52 kDa single-chain protein synthetized as a 418 amino acid precursor that migrates on SDS-PAGE as a single band with an approximate Mr of 52 kDa (lanes 2 and 3 of Figure 1, panel A). The AAT–HNE complex (Mr at about 80 kDa) is also shown in lanes 2 and 3 of the same panel.

The loss of the 24-aa signal peptide gives rise to a 394 amino acid mature protein containing three carbohydrate side chains linked by means of an N-glycosidic bond to three asparagine residues (Asn46, Asn83, and Asn247). Each carbohydrate side chain consists of N-acetylglucosamine, mannose, galactose, and sialic acid [3]. Several glycoforms of AAT exist, which differ for their properties in regard to plasma half-life and stability [11]. Glycosylation also plays an important role in determining the immune modulatory properties of AAT. It has been shown that alterations that occur to AAT glycans at times of inflammation (and in malignant conditions) may have an impact upon the functions of the protein [11]. AAT is mainly produced and secreted by hepatocytes and belongs to the superfamily of the Serine Protease Inhibitors (Serpins); these are encoded in humans by the *SERPINA1* gene, also known as the Pi gene, which is located on the long arm of the chromosome 14 (14q31–32.3).

Crystallographic studies on the mature protein demonstrated that AAT displays a nearly 360-residue structure for a serpin domain folded into 3 beta-sheets and 9 alpha-helices (see Figure 1, Panel B) [2]. AAT is synthesized in ribosomes associated to rough endoplasmic reticulum (RER), which is submitted to glycosylation and is correctly folded by exposing, in the upper pole of the molecule, the reactive center loop (RCL), containing the critical residue Met358 (see Figure 1). This domain is responsible for the bonding with HNE and its inhibition. The characteristic feature of AAT, that is the same for all serpins, is the so-called “suicide” inhibition mechanism occurring through a mousetrap route [2]. RCL contains 20 amino acids, 2 of which, indicated as P1-P1′ residues (Met358 and Ser359), are recognized by HNE that cleaves the peptide bond between these two residues, releasing the P1′ residue and the C-terminal peptide and forming an ester bond between HNE and AAT. Once the intermediate acyl-enzyme is formed, the RCL inserts as an extra strand in β-sheet A and, consequently, a transition from a “stressed to relaxed” conformation occurs by flipping the enzyme from the upper to the lower pole of AAT. The conformational change increases the number of strands in β-sheet A from five to six, increasing in turn the thermal stability of the serpin and stabilizing the acyl intermediate, since the diacylation of the complex is prevented [2].

**Figure 2:**
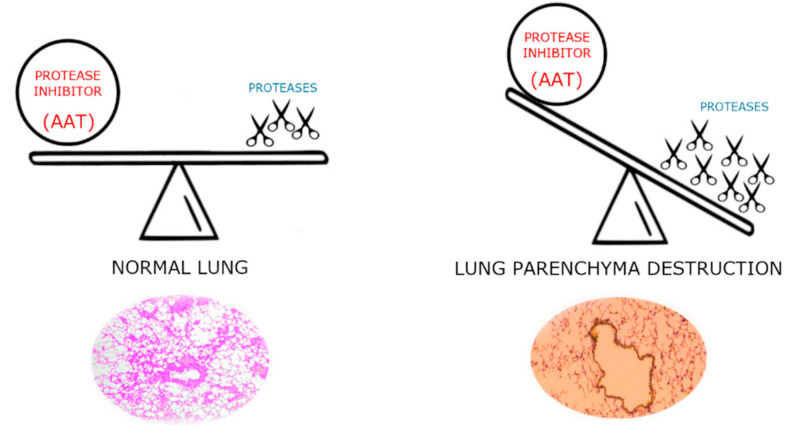
Mechanism of the imbalance between proteases and antiproteases leads to lung parenchyma destruction.

Although AAT displays a reference range of concentration in blood of 0.9–2.3 g/L, this value can rise many fold upon acute inflammation [11,12]. Despite this increase, due to the high amount of HNE produced under these circumstances, AAT cannot completely counteract it; consequently, it partially persists in the lung as free enzyme. As shown in the scheme of Figure 2, this imbalance between protease(s) degrading the parenchymal extracellular matrix and their inhibitors is responsible for the destruction of lung elastin and, if it is persistent or recurrent over years, it results in respiratory complications, such as chronic obstructive pulmonary disease (COPD) [2,3,4,5].

The preservation of the tertiary structure is essential, as both misfolding and the intracellular polymerization of SERPIN members lead to the onset of a variety of serpinopathies [13], among which α_1_-antitrypsin deficiency (AATD) is, most likely, one of the best characterized.

**Figure 3:**
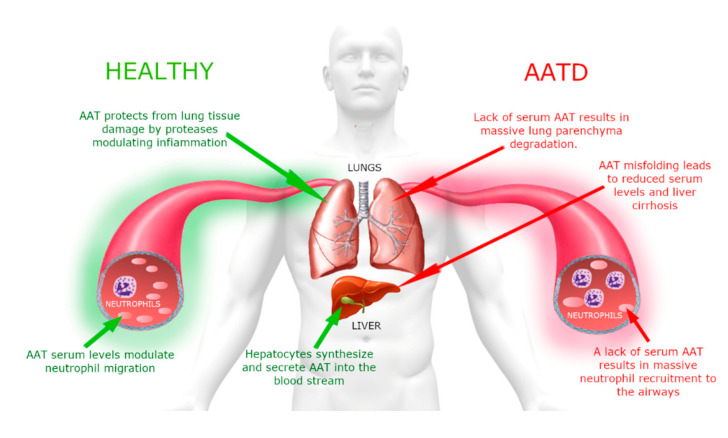
Schematic. representation of α1-antitrypsin deficiency (AATD) pathophysiology.

AATD is an autosomal co-dominant severe genetic disorder that affects lungs in adults and can be associated with liver disease in a small portion of affected children [14]. A schematic representation that summarizes the clinical aspects of this disorder is shown in Figure 3.

Although the exact AATD prevalence remains largely underestimated (being the diagnosis problematic due to the delayed onset of symptoms), it is believed to afflict millions of individuals worldwide [14,15,16].

There is currently no cure for this pathology, and the major goal of AATD management is slowing the progression of lung destruction. If the “conventional” treatments are essentially based on the use of bronchodilators, inhaled steroids, and antibiotics (when infections occur), augmentation (replacement) therapy, consisting of the intravenous infusion of the missing AAT protein, is presently being used, under special circumstances, as an efficient long-term medical treatment of AATD [17,18,19].

Based on the above considerations, AAT can be considered a “pharmaceutical form” that is administered as medication to AATD patients. Since AAT is typically purified from human blood, a remarkable impact on the biopharmaceutical industry has been caused by plasma shortage problems that have often disregarded the demand for a large-scale production of this protein. Consequently, the need to produce this therapeutic protein in a recombinant version has witnessed an enormous growth in recent years.

It is the authors′ intention to retrace in the following paragraphs of this article all technological advances that allowed scientists to move from a few micrograms of partially purified AAT to several grams of highly purified protein and to present its current applications in the clinical setting.

## 2. Methodology Used for Finding the Articles Used for the Preparation of the Current Review

A literature search of articles published between the 1960s and the present day was performed via the MEDLINE database. The following search terms were used: ‘Biochemical characteristics of Alpha-1 antitrypsin’; ‘Methods of purification of Alpha-1 antitrypsin’; ‘Recombinant Alpha-1 antitrypsin’; ‘Alpha-1 antitrypsin deficiency treatment’; ‘Augmentation therapy’.

Systematic reviews, as well as classical articles from the 1960s, were included for review. With the above-mentioned search terms, a total of 350 articles were found. The full text of these papers was analyzed and repeated articles and those with redundant content were eliminated. Finally, a total of 90 papers were selected.

## 3. Methods of AAT Purification: From the Beginning to the Present Day

Protein purification may range from a simple one-step (precipitation) procedure to complex approaches that involve more than one step. Although most purification schemes currently include several steps, the key to an efficient protein purification is to minimize the number of steps required. This is accomplished by selecting the most appropriate techniques, optimizing their performance and combining them in a logical sequence to maximize yield. Thus, the optimization of protein purification is a multifactorial exercise that requires specific responses to questions such as (i) which source is the most appropriate, (ii) how many components must be removed, (iii) what are the purity issues in relation to the starting material and the end-use of the product, and (iv) what will be the final scale of purification. Unfortunately, in the early 1950s, when interest in the purification of AAT started to grow (given the important physiological implications of this protease inhibitor), due to a variety of reasons, most of these questions could not be answered. First of all, the only AAT source practically used was human plasma, and second, the basic scenario of available methods was essentially restricted to protein precipitation by addition of ammonium sulfate. Finally, the use of this single procedure did not allow achieving the desired amount/purity level of protein. Not surprisingly, in fact, the results were poor in terms of both protein purity and quantity, the small amounts of AAT recovered being still heavily contaminated by interfering proteins. However, the aim of the researchers was to investigate the important biochemical features of AAT in view of its use as a therapeutic tool in reducing the tissue degradation caused by proteolytic enzymes. Thus, to reach the targets for yield and purity, additional strategies were requested. The emergence in the 1960s of chromatographic procedures turned out to be very advantageous in terms of the progression of AAT purification. The primitive process was complemented/replaced by powerful, sophisticated methods. All purification schemes currently used to purify AAT involve some form of chromatography. Used singularly or in combination, different chromatographic techniques with different selectivities have become attractive technological tools for the recovery of good amounts of highly purified material, suitable for therapeutic use.

The following paragraphs give a brief overview of the recent technological advances in AAT purification showing what has been the “historical” evolution of this process and how methodological choices may affect the physico-chemical characteristics of the final product.

### 3.1. Early Antecedents: Ammonium Sulfate Fractionation

As stated in the previous paragraph, one of the first attempts to isolate AAT from human plasma was performed by Duthie and Lorenz [20] in 1949. Little was known at the time about this protein, and only a few techniques were available. The conventional starting point was ammonium sulfate precipitation, a “capture step” that is useful to concentrate the protein and to remove some impurities that would be detrimental to purification efficiency. As the reader will see scrolling this article, although ammonium sulfate precipitation is a procedure for anything sophisticated, due to its effectiveness in the concentration process, it will be repeated up to the current times.

They added ammonium sulfate following the simple procedure developed a few years before by Schmitz [21]. The yield was very low (around 0.02%), and the serum inhibitor was only very partially purified. In the absence of an alternative approach, Peanasky and Laskowski [22] worked on the improvement of this fractionation procedure, and by slightly modifying the experimental conditions, they increased the yield to 5% and obtained a 50-fold purified protein. Essentially, Shulman et al. [23] also fractionated plasma proteins by the addition of ammonium sulfate. However, the addition of a further step consisting of filtration and the washings of precipitate with different solutions (i.e., 85% ethanol solution, ice-cold water, acetone) resulted in a dramatic improvement of the process. Surprisingly, they have been able to isolate this inhibitor not only from plasma but also from human urine in which AAT is much less abundant. This was the first report to mention the use of a biological fluid different from blood as an AAT source.

The common trait of all these approaches was the huge volume of fluids used as starting material i.e., up to 1000 L of urine or 10 L of plasma. Evaluated on the basis of the current criteria, it can wrongly be assumed that this “primordial” method was not very productive, the poor resolution being the most obvious characteristic.

However, if you look back, it is easy to see that despite their intrinsic limitations, these first efforts enabled the recovery of small amounts of active, although partially purified AAT, which allowed a better biochemical characterization of the protein.

All projects were not successful, nevertheless these attempts played a crucial role in pioneering the development of more sophisticated techniques that led to a large-scale production of AAT at a good level of purity.

### 3.2. The Introduction of Electrophoretic and Chromatographic Approaches

The logical evolution in AAT purification was the combination of the ammonium sulfate fractionation with other procedures that could take advantage of the physico-chemical properties of AAT. To make easier the separation of the large pool of proteins (typically the result of ammonium sulfate precipitation) into several smaller pools, one (or more) of which was enriched in AAT, the first parameter considered by investigators was the protein charge. The shape and charge on the protein surface are in fact well-known parameters that affect its binding to different matrices.

While Moll et al. [24] and Bundy et al. [25] explored the potential of ion exchange chromatography (on a Dowex resin), Myerowitz et al. [26] decided to combine chromatographic and electrophoretic techniques. This expedient allowed them to detect two immunologically distinct AAT forms in human serum and made this procedure very popular in the 1970s. This result was achieved by combining DEAE cellulose chromatography and affinity chromatography (to remove albumin) with preparative polyacrylamide gel electrophoresis.

Since the objective of all authors was to obtain the largest amount of the functional AAT with the fewest contaminants, the abundance of albumin in human plasma/serum was indeed the challenging obstacle to overcome. The role played by dye affinity chromatography in the specific removal of this protein is certainly worth emphasizing. The possibility to use albumin-free plasma as the starting material was a decisive step forward for AAT purification. It is no coincidence that all the authors mentioned in the continuation of this article have introduced dye affinity chromatography in their purification scheme.

This is what Whitley and Liener [27] did before applying preparative electrophoresis to successfully isolate AAT. This strategy turned out to be particularly interesting, since they developed and built an electrophoretic equipment of new formulation that allowed decreasing the length of the entire procedure and improving the yield of the inhibitor. A similar strategy was applied by Pannell et al. [28], who depleted plasma albumin using a column of Sepharose-Blue Dextran. Then, AAT was precipitated by adding ammonium sulfate (60–80% saturation) to the most active fractions from this step and further purified submitting the pellet to anion exchange chromatography on DEAE–cellulose. Disc electrophoresis and isoelectric focusing demonstrated the high degree of purity of this preparation [28].

A modification of Pannell’s procedure was applied by Sugiura et al. [29], who used DEAE–cellulose chromatography at pH = 6.6 followed by ammonium sulfate fractionation (50–80%) and DEAE–cellulose chromatography at pH = 8.8 as the first three steps. Sephadex G-100 and Con A Sepharose were the two steps that led to the final product whose homogeneity was checked by SDS electrophoresis.

In addition, Plancot et al. [30] purified AAT by applying a complex procedure that consisted of five steps, the first of which was ammonium sulfate precipitation. Then, the material was loaded on Blue Dextran Sepharose 4 B followed by preparative electrophoresis, Con A Sepharose, and DEAE–cellulose chromatography. At the end of this process, both N- and C-terminal analysis, SDS, and two-dimensional immune electrophoresis were established as criteria of homogeneity.

With hindsight, it would be easy to state that in the above cases, the authors did not observe the dogma “minimum number of steps and the simplest possible design” to reach the targets for yield and purity. However, while considering that AAT purification is not a simple job, it is obvious that the need to add one step to another (and repeat the same step) was strictly connected to the poor quality of chromatographic media available on the market in the 1970s that strongly determined the efficiency of the process.

Most likely to overcome these problems, Laurell et al. [31] isolated AAT from other plasma proteins adopt a strategy that greatly differed from the previous ones. They conjugated monomeric χ-chains with cyanogen bromide (CNBr)-activated Sepharose 4B to perform thiol–disulfide interchange chromatography. This interchange reaction was greatly facilitated if the terminal χ-chain cysteine exists in the form of a mixed disulfide; 5,5-dithio-bis (2-nitrobenzoate) was used to form it. As demonstrated by agarose gel electrophoretic analysis, this approach did not exert any detectable effect on the microheterogeneity of AAT. The protein was successfully purified from dog, baboon, and monkey.

The original thiol-exchange method was also applied by Vaughan et al. [32], who isolated the AAT isoforms with the purpose of investigating in depth the microheterogeneity of the protein

### 3.3. The Structural Combination of Different Chromatographic Techniques

The success in the purification level observed with the advent of a wide range of sophisticated materials in the ion-exchange and affinity chromatography fields was slow but steady and encouraging. Although the purification strategy was not completely transformed (the first step, in most cases, still being ammonium sulfate precipitation), by taking advantage of the peculiar features of these novel matrices, researchers explored the effects of their combination in planning purification schemes of AAT that would otherwise be time-consuming. This allowed obtaining the protein under the most stabilizing conditions and to maximize the yield. The following examples attest to the progress that has been made in this difficult field.

One of the first significant applications of these techniques was that of Morihara et al. [33], who purified the inhibitor by modifying a previously developed method [34]. In brief, after saturating human plasma with ammonium sulfate (80%), the pellet was dissolved in phosphate buffer pH 8.0, dialyzed, and loaded on an Affi-GEL Blue column. Two subsequent steps on a Zn-chelate column followed by a DE-ion exchange chromatography allowed producing homogeneous AAT. The purified protein was used to clarify the mechanism of AAT inactivation by *P. aeruginosa* elastase.

An intriguing exercise in which the differences in interaction strengths between the different biomolecules within a mobile phase and the stationary phase were exploited was done by Dubin et al. [35]. They have been able to purify nine plasma proteinase inhibitors, including AAT, from a single batch of human plasma, using a set of seven different affinity chromatographic columns connected in series. The whole process for AAT required a further gel filtration step that was not necessary for other inhibitors.

The growing popularity captured by the chromatographic media led Finette et al. [36] to explore the optimum operational performance of silica-based ion-exchange adsorbents in packed and expanded beds for the purification of AAT. This study was focused on the development of a technique for separating AAT from serum albumin alternative to the ethanol plasma fractionation procedure previously published by Cohn et al. [37]. Working on Cohn Fraction IV (the plasma fraction that contains about 33% of AAT, used by several authors as an AAT source), they investigated the chemico-physical properties of the anionic exchanger DEAE–Spherodex LS. The influence of fluid superficial velocity on the bed dispersion number was verified, and the dispersion coefficient was determined. In addition, the consequences of changes in a wide number of physical parameters (i.e., superficial velocity, feedstock protein concentration, loading volume duration, frequency of the wash cycle, and extent of bed expansion) on the adsorption capacity and relative purification factors of AAT present in the serum were also explored. The results of these investigations showed that the flow rate and the extent of column utilization affected to different extents the AAT yield and the serum albumin recovery. An increase of both the column loading and the flow rate reduced the AAT yield. Taken together, these results have greatly improved the knowledge of the drawbacks encountered during the purification process of AAT.

The purification of AAT from Cohn Fraction IV was described also by Chen et al. [38], who followed an articulated procedure consisting of a combination of complementary chromatographic steps. They coupled anion to cation exchange chromatography used as the first and second step, respectively. Then, the mixture was submitted to tri-*n*-butyl cholate treatment (to inactivate enveloped viruses) and to a further cation chromatography step. The whole recovery of AAT was around 64–70%, and its purity was above 95% by SDS analysis.

The AAT source chosen by Kumpalume et al. [39] was the soluble fraction (A+I supernatant) obtained after treating plasma with 19% ethanol at pH 5.85 according to the method of Kistler and Nitschmann [40]. The novel method they developed combined ion exchange with two metal–chelate chromatographic processes. In addition, in this case, the yield was around 60%, and the purity of the protein was above 90%.

Given the importance of using AAT as a drug for the potential cure of lung emphysema induced by congenital AAT deficiency, a strategy for the isolation of large amounts of highly pure AAT was evaluated by Zhang et al. [41]. This goal was reached by specifically removing (by means of silica) α1-lipoprotein from Cohn Fraction IV and submitting the collected material to immunoaffinity chromatography (AAT Select was used as ligand) on a fast-protein liquid chromatographic (FPLC) system. Thanks to the availability of highly advanced analytical approaches (SDS-PAGE followed by Western blotting; two-dimensional electrophoresis, capillary electrophoresis, electrospray ionization-tandem mass-spectrometry, and database searching) the product they obtained was 100% pure and characterized by the highest possible specific activity (1.00–1.05 Plasma Units/mg).

Instead of ammonium sulfate, Huangfu et al. [42] tested the potential of polyethylene glycol (PEG), a nondenaturing water-soluble polymer, to alter the solubility of AAT from that of other proteins present in Cohn Fraction IV. The PEG concentration required to obtain protein precipitation is well-known to be dependent on the size of both protein and polymer, being unique for a given protein–polymer pair. The concentration used to manipulate the solubility of AAT and obtain its precipitation was 15% PEG 4000. The manufacturing process included the utilization of anion-exchange chromatography combined with two steps of Blue Sepharose affinity chromatography. The specific activity (3893.29 IU/mg) of AAT was even higher than that of some of the current commercially available AAT therapeutic products. These experimental conditions, suitably optimized, allowed the authors to produce AAT on a large scale [43].

Another procedure noteworthy for its peculiarity is that adopted by Fan et al. [44], who explored the potential application of membrane chromatography in the plasma products industry. Human plasma fraction IV precipitate was dissolved in an appropriate buffer and lipoproteins were removed by PEG precipitation. The surnatant was first submitted to anion-exchange membrane chromatography (AEMC), which is a peculiar technology where the transport of solutes to their binding sites takes place mainly by convection. As underlined by the authors, the usefulness of this mechanism consisted in a decrease of both process time and buffer consumption. The AAT purity level was further improved by hydrophobic interaction membrane chromatography (HIMC). All experimental conditions (including pH, ionic strength, gradient slope, flow rate, and loading volume) were systematically optimized and, to verify the efficiency of the membrane-chromatography approach, the results were compared with those obtained by using the corresponding column-chromatographic procedures. This data-matching demonstrated that while the recovery and purity of AAT from both AEMC and the corresponding chromatographic process were comparable, those obtained by HIMC were even better than the corresponding chromatographic ones.

### 3.4. The Production of Recombinant AAT

Until a few years ago, the only source to support the therapies for the diseases mentioned in the above paragraphs was plasma AAT. However, as underlined in previous paragraph, due to recurrent plasma shortage (limited supply of donated blood), the frequent difficulty of native AAT to meet the market demand caused a remarkable impact on the biopharmaceutical industry. Consequently, the need to produce this therapeutic protein via recombinant routes became urgent. The remarkable scientific advances of the last decades, which made protein overexpression and purification quite easy, allowed this alternative way to witness an enormous growth that resulted in the development of different production routes to be alternative/join the conventional eukaryotic systems. Obviously, once the problem relative to the production of good amounts of AAT was solved, the second key issue addressed by researchers was that of its quality. In fact, as previously stated, AAT is a glycosylated protein in which the level of glycosylation plays important roles in modulating the plasma half-life/stability and the immune modulatory properties of the protein. Thus, as plant cell cultures offer a number of advantages over other expression systems (i.e., the possibility to purify the product from the cell culture broth), a major question for the authors suggesting the use of transgenic plants as source of recombinant AAT was the level of glycosylation of the protein [45,46].

Among the numerous reports that attest to the feasibility of several approaches in this area, the most interesting will be presented in this section.

Kwon et al. [47] were forerunners in the purification from yeast of recombinant AAT that was secreted in the medium as a glycosylated form. They developed a procedure that involved the precipitation of the protein with ammonium sulfate (60–75% saturation) followed by a series of subsequent chromatographic steps consisting of anion exchange (DEAE and mono Q columns) and affinity (Affi-Gel Blue column) chromatography. Although the yeast-produced AAT was fully functional as a protease inhibitor (compared with the plasma form), the molecular mass of this protein (unlike plasma AAT), once treated with endoglycosidase H, decreased to that of recombinant AAT produced in *Escherichia coli.* This indicated that the N-linked glycosylation of this form was high mannose-type. The authors also observed that glycosylation conferred the yeast-produced AAT an enhanced kinetic stability toward heat inactivation.

Recombinant AAT with a modified synthetic gene was expressed in transgenic tomato plants at a very high level (3.2% of total soluble proteins) by Jha and Agarwal [45]. The ammonium sulfate precipitation step (90% saturation) was followed by several chromatographic techniques including hydrophobic interaction chromatography, anion exchange chromatography, affinity chromatography, and finally, immunoaffinity chromatography on a column prepared by coupling rabbit anti-human AAT polyclonal antibody to CNBr-activated Sepharose 4B matrix. The excellent specificity of this step allowed obtaining a protein that exhibited molecular mass of about 48 kDa and a pI of about 5.1. As confirmed by sophisticated analyses (SDS-PAGE and Western immunoblotting together with MALDI-TOF and circular dicroism spectroscopy), the plant-purified AAT and the native serum protein showed a strict similarity in secondary structure and molecular mass. The difference in molecular size (about 4 kDa) between the two proteins may be attributed to the differential glycosylation pattern in the recombinant one, particularly for the absence of terminal galactosylation and sialylation. Moreover, when produced by plants, glycosylated proteins display a non-human pattern of polysaccharides, with subsequent interaction with natural antibodies and shorter half-life. The tomato plant-purified AAT clearly demonstrated effective binding and the formation of a stable complex with elastase in an equimolar ratio. 

Zhang et al. [46] successfully established a pilot purification protocol for the large-scale production of AAT for antitrypsin therapy expressed in transgenic rice (*Oryza sativa*) endosperm. The powder obtained from brown rice was extracted, and the supernatant was loaded on a DEAE column. The eluted material was applied to a column containing a mixed-mode support of ceramic hydroxyapatite followed by a multimodal strong anion exchanger. Affinity chromatography performed on a Con A column was the last step that allowed producing 2.24 g of AAT (with a 97% purity) per kg of brown rice. Several analytical tests (circular dichroism, isoelectrofocusing, N-terminal, and MALDI–TOF analyses) demonstrated that the biochemical characteristics and biological activity of recombinant protein were equivalent to those of plasma AAT, both being heterogeneous proteins with the diversity of glycan modification and the absence of the N-terminal peptide. These results allowed the authors to demonstrate the potency of rice endosperm as a promising system to express a protein with biopharmaceutical interest.

Due to the high production capabilities of the mammary gland, also the prospect of producing large quantities of AAT in the milk of transgenic livestock has attracted a great appeal. Considering the simplicity of accessing the expressed protein and the relatively low operating costs, this was suggested as a simple and efficient expression system for recombinant AAT [48].

From the fusion of the ovine β–lactoglobulin gene promoter to the human AAT genomic sequences, Wright et al. [48] have been able to obtain human AAT (hAAT) from the milk of transgenic sheep. Milk was defatted and hAAT was purified from the remaining material using, sequentially, anion exchange, dye affinity, hydrophobic interaction, and gel-filtration chromatography. Based on its electrophoretic mobility, the apparent molecular size of the material purified from the transgenic sheep milk suggested that it was completely glycosylated, although the nature of the sugar moieties was not determined. In addition, the ability of the recombinant protein to inhibit trypsin was very similar, if not identical, to that of plasma-derived AAT.

Taken together, the data from SDS-PAGE; HPLC- and N-terminal analyses did not reveal any contamination with sheep AAT and showed that the transgenic protein was >95% pure.

Kang et al. [49] evaluated the signal sequence derived from inulinase (INUIA) of *Kluyveromyces marxianus* in directing the secretion of AAT from *Saccharomyces cerevisiae.* A yeast expression vector for AAT was constructed by placing the coding sequence of human AAT fused with the INUIA signal sequence downstream of the *GAL10* promoter. About 70% of the total human AAT synthesized in the recombinant yeast was secreted into the extracellular medium by the inulinase signal peptide. The majority of intracellularly-retained AAT was unglycosylated, suggesting that once the human AAT enters into the yeast ER and is glycosylated, the protein is very rapidly transported through the secretion pathway to the outside cells. The secreted protein had high mannose-type glycosylation.

Proteins present in the culture supernatant were precipitated by the addition of ammonium sulfate (80% saturation); the pellet was collected, dissolved, and applied to a DEAE–Sephacel column. Fractions containing AAT activity were pooled and further fractionated by Con A agarose chromatography. Affi-Gel Blue and Mono Q HR were the last two chromatographic steps that allowed obtaining the highly purified protein.

These data demonstrated that the yeast system using the *GAL10* promoter and the inulinase signal sequence is a useful tool to produce large quantities of correctly folded human AAT protein into culture media.

The methods described, together with the yield of AAT recovered, the level of purity, the fold purification, and the reference number of the related article have been schematized in Table 1.

## 4. Application of AAT: Augmentation Therapy and Its Alternatives

Since 1987, AAT augmentation therapy is being used in the treatment for AATD in some countries. This therapy involves the intravenousadministration to the patients of plasma-purified human AAT to normalize their AAT serum concentration to above the protective threshold of 11 µM. Interesting considerations about the goals of this therapy (i.e., re-establishing physiological concentrations of AAT; preventing/slowing and modifying the course of disease progression; reducing the frequency of exacerbations and improving life quality) as well as of the clinical experience gained in its use for AATD during the past 25 years are finely presented in a variety of excellent reviews by different authors [19,50,51,52,53,54].

An alternative route to intravenous injections is the aerosol therapy, also known as inhaled AAT therapy, which has the major advantage of not requiring an intravenous access. While not being a big problem when AAT is administered for brief periods, the risk of thrombosis and infection is high when repeated intravenous accesses with weekly administration for many years (as recommended for AATD) are needed. This inhaled therapy entails the inhalation of human plasma-derived AAT, which is directly deposited into the lung.

An exciting review article focused on this topic was published by Usmani O.S. [55]. The author shows that the inhaled route allows easier delivery to the lungs of AAT that reaches the target site of action with higher levels. Moreover, the treatment is not at all invasive, and patients can administer it alone without traveling to specialized units. The opposite site of this medal is represented by the ability of inhalation to target good amounts of drug to the emphysematous lung parenchyma of the small alveolated airways, i.e., the pathophysiological site of disease. However, technological and chemical advances in the fields of nebulizers and formulation science, respectively, resulted in the consistent and reproducible delivery of adequate doses of AAT to the epithelial lining fluid of the small airways. Results from a placebo-controlled clinical trial have shown that this treatment with nebulized AAT was safe and achievable in a large patient cohort.

In addition to the above cited reviews, other interesting articles focused on some of the highlights arising from the research on AAT augmentation therapy.

A randomized controlled trial (RCT) was carried out by Chapman et al. [56] on 180 non-smokers (aged 18–65 years) recruited in 28 international study centers in 13 countries to evaluate the clinical effectiveness of this therapy. The aim of this two-year randomized, double-blind, placebo-controlled (RAPID-RCT) study was to compare the effects on these subjects of an augmentation therapy, which was administered intravenously at 60 mg/kg of body weight each week, to placebo infusions. The primary outcome of this study, as assessed by CT, was a statistically significant annual reduction of the rate of lung density decline in the treated group. Secondary outcomes included forced expiratory volume in one second (FEV1) and AAT concentrations. The results of this study demonstrated a slight but debatable significant change in lung density in the long term, although lung function improvement was undetectable. The finding that the AAT levels were significantly increased suggested that the administered AAT was not immediately degraded.

During a meta-analysis carried out by the same group [57] on 1509 AATD patients, the hypothesis was tested that augmentation therapy could slow the FEV1 rate of decline. The results of this analysis clearly demonstrated that the rate of loss of lung function was slower by about 25% among all patients receiving augmentation therapy. These encouraging data supported the conclusion that AAT supplementation can slow lung function decline in patients with this deficiency and that subjects with moderate obstruction are most likely to benefit.

In the open-label extension trial (RAPID-OLE, a follow-up study), McElvaney et al. [58] surveyed the participants of the previous RAPID-RCT study for two additional years. While confirming the results of this latter study, the authors evidenced a significant inflection point in lung density loss for patients who started the AAT therapy at the start of the RAPID-OLE trial. The authors concluded that the experimental evidence supported the continued efficacy of augmentation therapy in slowing disease progression during 4 years of treatment. The observation that lost lung density was never recovered highlighted the importance of early intervention with this treatment.

Campos et al. [59] showed in their study that subjects with AATD on standard dose (SD; 60 mg/kg/wk) augmentation therapy still exhibited inflammation, protease activity, and elastin degradation. They evaluated the biological effects of normalizing AAT levels in these subjects with double-dose (DD) therapy (120 mg/kg/wk). The results were encouraging, since serine protease activity in bronchoalveolar lavage fluid (BALF) (elastase and cathepsin G) as well as elastin degradation (desmosine/isodesmosine) in BALF were reduced. They concluded that higher AAT dosing than currently recommended should be explored further to understand better whether it may lead to enhanced clinical benefits.

Although the outcomes of the studies above discussed apparently prove the efficacy and effectiveness of AAT augmentation therapy, this argument is questioned by some authors [60,61] who argue that, due to several inconsistencies or flaws of these works (i.e., confounding factors not considered, weaknesses in the methodology used, and high cost of the treatment), the evidence provided by the current data do not sufficiently support this conclusion.

As a result of this dispute and of the fact that the effect of the AAT augmentation therapy on the protease–antiprotease balance is still incompletely understood, the treatment has only been implemented in a few countries [61].

Nevertheless, various studies into different types of AAT therapy are being carried out.

Gene therapy is another alternative that is being investigated using different methods of altering gene expression. One of these is transfecting the M-AAT gene to the patients by means of a vector. Currently, different clinical trials are being performed to discover the best administration route. The findings by Mueller et al. [62] suggest that muscle-based AAT gene replacement is tolerogenic and that stable levels of M-AAT may exert beneficial effects increasing serum anti-elastase activity.

Other promising approaches involving gene transfection that are being explored are the use of a transposon to correct the point mutations in SERPINA1. In a very appealing article, Yusa et al. [63] show that a bi-allelic correction of a point mutation (Glu342Lys) in Human induced pluripotent stem cells (hIPSCs) of the AAT gene responsible for AATD restored the structure and function of AAT in subsequently derived liver cells in vitro and in vivo. This was the first proof of principle that the combination of hIPSCs with genetic correction has the potential to generate clinically relevant cells for autologous cell-based therapies. According to the authors, this approach was “significantly more efficient than any other gene-targeting technology that is currently available and crucially prevents contamination of the host genome with residual non-human sequences”.

Finally, in a recent article, Turner et al. [64] evaluated the use of siRNA to interfere with and degrade the mRNA of Z-AAT. The results of their study demonstrated that both PiZZ patients and healthy volunteers responded similarly to ARC-AAT and based on an observed reduction in serum AAT concentrations, that subjects showed a deep and durable knockdown of hepatic AAT production.

As the studies into these therapies are all still in an early phase, implementation will probably take a while.

## 5. Conclusions

Alpha1-antitrypsin deficiency (AATD) is a genetic disorder characterized by increased risk for developing both early-onset lung emphysema and chronic liver disease due to the reduced serum levels of alpha1-antitrypsin (AAT). To protect the lungs from the proteolytic damage caused by proteases, replacement (also indicated as augmentation) therapy with the missing AAT protein is an innovative treatment for this condition that may be used under special circumstances. While being particularly attractive, this treatment requires the production of good amounts of highly purified AAT from human plasma. It is apparent that this process would benefit from the availability of procedures efficient enough for the routine production of this precious material. This article aims to provide a critical review of the methodological steps that have marked progress in the purification of this protease inhibitor. It has been planned to provide an overview of the literature concerning the strategies developed over the years in AAT production and the most recent practical applications. The numerous publications discussed in this report underline the enormous progression achieved in these years regarding the laboratory procedures developed for the purification of AAT. The jump from small quantities of raw material to large amounts of highly purified protein was achieved thanks to the improved experimental design/sample preparation together with the development of novel platforms. Nevertheless, the constantly growing appearance on the market of excellent chromatographic materials and the rapid pace of technological advances have also given impetus to explore the use of increasingly efficient techniques. Taken together, all these implementations have attracted increasing attention from numerous groups, and this has greatly facilitated the efforts devoted to this research. A significant impact in this area is still expected in coming years.

Since AATD is classified as a rare disease (the prevalence is 1–5/10,000), some may question the need for such comprehensive efforts put in a direction that benefits relatively few patients. However, in our opinion, this view is short-sighted, since it seems plausible that efforts to define better the pathogenesis of AATD, by studying in depth the effects of augmentation therapy, may serve to gain further insight also into other correlated disorders. Thus, the application of sophisticated techniques capable of purifying AAT at a very high level and/or of producing this protein from sources different from human plasma is expected to make the improvement of knowledge regarding this disease easier.

## Figures and Tables

**Table 1:** List of methods described in the text indicating: (i) the source of AAT, (ii) the yield of AAT recovered, (iii) the level of purity, (iv) the fold purification, and (v) the reference number of the related article.

Source	Method Used	Yield % *	Purity % **	Fold Purification	Reference #
Human serum	Ammonium sulfate fractionation	0.02	≈20	n.r.*	[20]
Human serum	Ammonium sulfate fractionation	5	≈20	50	[22]
Human urine Human serum	Ammonium sulfate fractionation Washings of precipitate with different solutions (i.e., 85% ethanol solution, ice-cold water, acetone)	10	≈20	n.r.*	[23]
Human serum	Ammonium sulfate fractionation Anion exchange chromatography	37.6	≈50	21	[24]
Human plasma	Ammonium sulfate fractionation Anion exchange chromatography	4	≈70	67	[25]
Human serum	Ammonium sulfate fractionation DEAE cellulose chromatography Affinity chromatography Preparative polyacrylamide gel electrophoresis	35	≈70	87	[26]
Human serum	Affinity chromatography Preparative polyacrylamide gel electrophoresis	70	≈90	90	[27]
Human plasma	Blue Dextran Sepharose chromatography Ammonium sulfate fractionation DEAE cellulose chromatography	60	≈90	n.r.*	[28]
Human serum	DEAE cellulose chromatography (pH 6.6) Ammonium sulfate fractionation DEAE cellulose chromatography (pH 6.6) Gel filtration (Sephadex G-100) Con A chromatography	85	≈90	93	[29]
Human serum	Immunoadsorbent column chromatography	85	≈90	90	[29]
Human serum	Ammonium sulfate precipitation Blue Dextran Sepharose 4B chromatography Preparative gel electrophoresis Con A Sepharose DEAE–cellulose chromatography	15	≈90	35	[30]
Human, baboon, monkey and dog plasma	Thiol Sepharose chromatography Thiol–disulfide interchange chromatography	50	≈90	95	[31]
Human plasma	Thiol Sepharose chromatography	n.r.*	≈90	n.r.*	[32]
Human plasma	Ammonium sulfate precipitation Affi-GEL Blue column Zn-chelate column DE-ion exchange chromatography	21	≈90	4	[33,34]
Human plasma	Seven affinity chromatographic columns connected in series Gel filtration chromatography	56	≈90	n.r.*	[35]
Cohn Fraction IV	DEAE–Spherodex LS	37	≈90	60	[36]
Cohn Fraction IV	DEAE chromatography Cation chromatography Tri-*n*-butyl cholate treatment Cation chromatography	67	≈95	61	[38]
A+I supernatant	Capto Q anion exchange chromatography NTA Sepharose Chelating Sepharose	60	≈90	30	[39]
Cohn Fraction IV	Immunoaffinity chromatography Fast-protein liquid chromatography (FPLC)	14	≈95	72	[41]
Cohn Fraction IV	Polyethylene glycol (PEG) precipitation Anion-exchange chromatography Blue Sepharose affinity chromatography	28	≈95	65	[42,43]
Human plasma fraction IV	PEG precipitation Anion-exchange membrane chromatography (AEMC) Hydrophobic interaction membrane chromatography (HIMC)	83	≈90	15	[44]
Transgenic tomato (*Solanum lycopersium*)	Ammonium sulfate precipitation Hydrophobic interaction chromatography Anion exchange chromatography Affinity chromatography Immunoaffinity chromatography	54	≈90	84	[45]
Transgenic rice (*Oryza sativa*) endosperm	DEAE–chromatography Ceramic hydroxyapatite (CHT) column Multimodal strong anion exchange chromatography Affinity chromatography	19	≈95	n.r.*	[46]
Recombinant yeast	Ammonium sulfate fractionation Anion exchange chromatography (DEAE and mono Q columns) Affinity chromatography (Affi-Gel Blue column)	16	≈90	95	[47]
Milk from transgenic sheep	Anion Exhange chromatography Dye cromatography Hydrophobic interaction chromatography Gel-filtration chromatography	50	≈90	98	[48]
Transgenic *Saccaromyces cerevisiae*	Ammonium sulfate precipitation DEAE–chromatography Con A agarose chromatography Affi-Gel Blue chromatography Mono Q HR chromatography	70	≈95	n.r.*	[49]

* Calculated on the basis of protein concentration. ** Not reported.
